# Genome sequencing identifies complex structural *MLH1* variant in unsolved Lynch syndrome

**DOI:** 10.1002/mgg3.2151

**Published:** 2023-02-09

**Authors:** Dennis Witt, Ulrike Faust, Gertrud Strobl‐Wildemann, Marc Sturm, Rebecca Buchert, Theresia Zuleger, Jakob Admard, Nicolas Casadei, Stephan Ossowski, Tobias B. Haack, Olaf Rieß, Christopher Schroeder

**Affiliations:** ^1^ Institute of Medical Genetics and Applied Genomics University Hospital Tübingen Tübingen Germany; ^2^ MVZ Humangenetik Ulm Ulm Germany; ^3^ NGS Competence Center Tübingen Tübingen Germany

**Keywords:** copy‐number neutral, genome‐sequencing, HNPCC, Lynch syndrome, *MLH1*, structural variant

## Abstract

**Background:**

Lynch syndrome is one of the most common cancer predisposition syndromes. It is caused by inherited changes in the mismatch repair pathway. With current diagnostic approaches, a causative genetic variant can be found in less than 50% of cases. A correct diagnosis is important for ensuring that an appropriate surveillance program is used and that additional high‐risk family members are identified.

**Methods:**

We used clinical genome sequencing on DNA from blood and subsequent transcriptome sequencing for confirmation. Data were analyzed using the megSAP pipeline and classified according to basic criteria in diagnostic laboratories. Segregation analyses in family members were conducted via breakpoint PCR.

**Results:**

We present a family with the clinical diagnosis of Lynch syndrome in which standard diagnostic tests, such as panel or exome sequencing, were unable to detect the underlying genetic variant. Genome sequencing in the index patient confirmed the previous diagnostic results and identified an additional complex rearrangement with intronic breakpoints involving *MLH1* and its neighboring gene *LRRFIP2.* The previously undetected structural variant was classified as medically relevant. Segregation analysis in the family identified additional at‐risk individuals which were offered intensified cancer screening.

**Discussion and Conclusions:**

This case illustrates the advantages of clinical genome sequencing in detecting structural variants compared with current diagnostic approaches. Although structural variants are rare in Lynch syndrome families, they seem to be underreported, in part because of technical challenges. Clinical genome sequencing offers a comprehensive genetic characterization detecting a wide range of genetic variants.

## Graphical abstract

A family with Lynch syndrome is presented by this article. Standard diagnostic tests, such as panel or exome sequencing, were unable to detect the underlying copy‐number neutral structural genetic variant in MLH1. This case aims to illustrate the advantages of clinical genome sequencing in routine diagnostics in detecting structural variants.

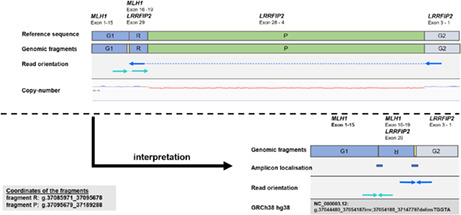

## 1 BACKGROUND

Lynch syndrome (LS) is one of the most common cancer predisposition syndromes and is associated with an increased risk for colorectal, endometrial, ovarian, urothelial, and other cancers (Dominguez‐Valentin et al., 2020; Engel et al., 2006; Seppala et al., 2021). LS follows the autosomal‐dominant inheritance pattern and is caused by pathogenic and likely pathogenic (P/LP) variants in four genes of the DNA‐Mismatch repair pathway (*MLH1* (OMIM *120436), *MSH2* (OMIM *609309), *MSH6* (OMIM *600678), and *PMS2* (OMIM *600259)) and by deletions in *EPCAM* (OMIM *185535) (Tutlewska et al., 2013). Deletions of *EPCAM* lead to an epigenetic silencing of the neighboring gene *MSH2* (Tutlewska et al., 2013). It is estimated that around 3% of colorectal cancer patients have LS and that LS explains approximately 10–25% of all familial colorectal cancers (CRCs) (Lynch et al., 2009). Cancer screening and surveillance are offered to index patients as well as at‐risk family members (Seppala et al., 2021). Previous studies have shown the effectiveness of special screening programs in LS patients (Lindor et al., 2006; Yurgelun & Hampel, 2018). Thus, an early and efficient genetically confirmed diagnosis is the first step for LS patients when they enter into screening programs.

The diagnosis of LS is based on family history (e.g., according to the Amsterdam or Bethesda criteria), histopathological findings, and germline and somatic genetic test results (Seppala et al., 2021). Defects in the MMR pathway lead to microsatellite instability (MSI‐H) and loss of protein expression of MMR proteins in immunohistochemistry (IHC). If a loss of *MLH1* and *PMS2* is detected by IHC in tumor tissue, the additional presence of a *BRAF* V600E variant in tumor tissue or of promotor methylation of *MLH1* likely indicates a sporadic CRC case. However, a positive *BRAF* V600E variant does not completely rule out the presence of HNPCC syndrome, especially for younger patients (< 50 years of age) (Blaker et al., 2020). Subsequent germline testing includes NGS panel sequencing of the coding regions of *MLH1/PMS2* and *MSH2/MSH6*, as well as checking for deletions containing exon 9 of *EPCAM*. Diagnostic approaches today mainly use targeted panel sequencing to identify small coding or splicing variants combined with MLPA for gross deletions or duplications. It should be noted that *PMS2* sequencing is a challenge from a technical standpoint. This is due to the presence of a large family of highly homologous *PMS2* pseudogenes, especially of *PMS2CL*. Molecular approaches utilizing longer DNA fragments, such as long‐range PCR (LR‐PCR), can amplify *PMS2* specifically, but not the pseudogenes (Herman et al., 2018).

A P/LP variant in the MMR genes is found in approximately 50% of all families fulfilling the Amsterdam‐II criteria (Park et al., 2002). In families that meet the revised Bethesda or Amsterdam‐II criteria and have a noticeable finding in the microsatellite analysis and IHC‐based tumor examination, germline variants can be found in about 60% of patients (Engel et al., 2006). The genetic background of the remaining cases remains unclear. As genome sequencing (GS) becomes a routine diagnostic test, it is expected that variants in intronic or regulatory regions, as well as structural variants, are identified more frequently and help to solve additional cases, as these variants are vastly undetected by today's standard approaches (Arnold et al., 2020; Morak et al., 2011).

GS has proven superior in terms of diagnostic sensitivity in other disease entities (e.g., intellectual disability). This is supported by systematic reviews regarding the superiority of ES and GS for detecting causative variants in cancer predisposition genes (Rotunno et al., 2020). For genetic sequencing of hereditary pediatric syndromes, an improved yield of almost 100% compared with single gene testing was reported (Lionel et al., 2018). The special capabilities of GS lie in its detection of structural and intronic variants (Cheng et al., 2016).

Here, we report an Amsterdam‐II criteria‐positive family who has suspicious tumor test results but have not had a molecular diagnosis for over a decade. While it was not possible to find the underlying cause via panel sequencing, MLPA, and exome sequencing, we identified a causal structural variant in *MLH1* by genome sequencing. This case illustrates the benefit of GS in a diagnostic setting by the inclusion of intergenic and intronic regions.

### 1.1 Ethical compliance

Written informed consent was obtained from all probands, and the research performed in this study was approved by the ethics board of the University of Tübingen (project number 066/2021BO2).

### 1.2 Case presentation

The reported individual (III‐4) is a male Caucasian who was referred to our outpatient clinic at the age of 55 years. He was diagnosed with simultaneous colorectal and bile duct cancer at the age of 52 years.

Amsterdam‐II criteria for HNPCC/LS were fulfilled in his family (Table 1). Both cancers were identified as adenocarcinomas. IHC of the MMR proteins and microsatellite analyses in CRC tissue revealed a loss of *MLH1* and *PMS2* expression and microsatellite instability. Neither *BRAF* V600E‐mutation nor *MLH1*‐promotor methylation were identified in tumor tissue. Follow‐up panel‐based NGS, in combination with MLPA for copy‐number analysis, failed to detect a causal variant.

**TABLE 1:** Anamnesis of family members.

Proband	Age	Died	Cancer #1 (age of diagnosis)	Cancer #2 (age of diagnosis)	IHC (loss of MMR)
I‐1	80	Yes	/	/	Not performed
I‐2	40	Yes	Uncertain (30–40 years)	/	Not performed
II‐1	93	No	/	/	Not performed
II‐2	50	Yes	Gastric cancer (48)	Colorectal cancer (48)	Not performed
III‐1	59	No	/	/	No Loss
III‐2	64	Yes	Gastric cancer (61)	/	*MLH1/PMS2*
III‐3	51	Yes	Klatskin tumor (51)	Endometrial cancer (51)	*PMS2*
III‐4*	55	No	Colorectal cancer (52)	Biliary tract cancer (52)	*MLH1/PMS2*
III‐5	53	No	/	/	Not performed
IV‐1*	40	No	Squamous cell carcinoma (40)	Solar keratosis (40)	No Loss
IV‐2	41	No	/	/	Not performed
IV‐3*	32	No	/	/	Not performed
IV‐4	30	No	/	/	Not performed
IV‐5	37	No	/	/	Not performed

*Note*: This table presents the families' anamnesis as collected in genetic counseling. Subjects tested for the identified *MLH1* variation are marked by an asterisk.

**FIGURE 1:**
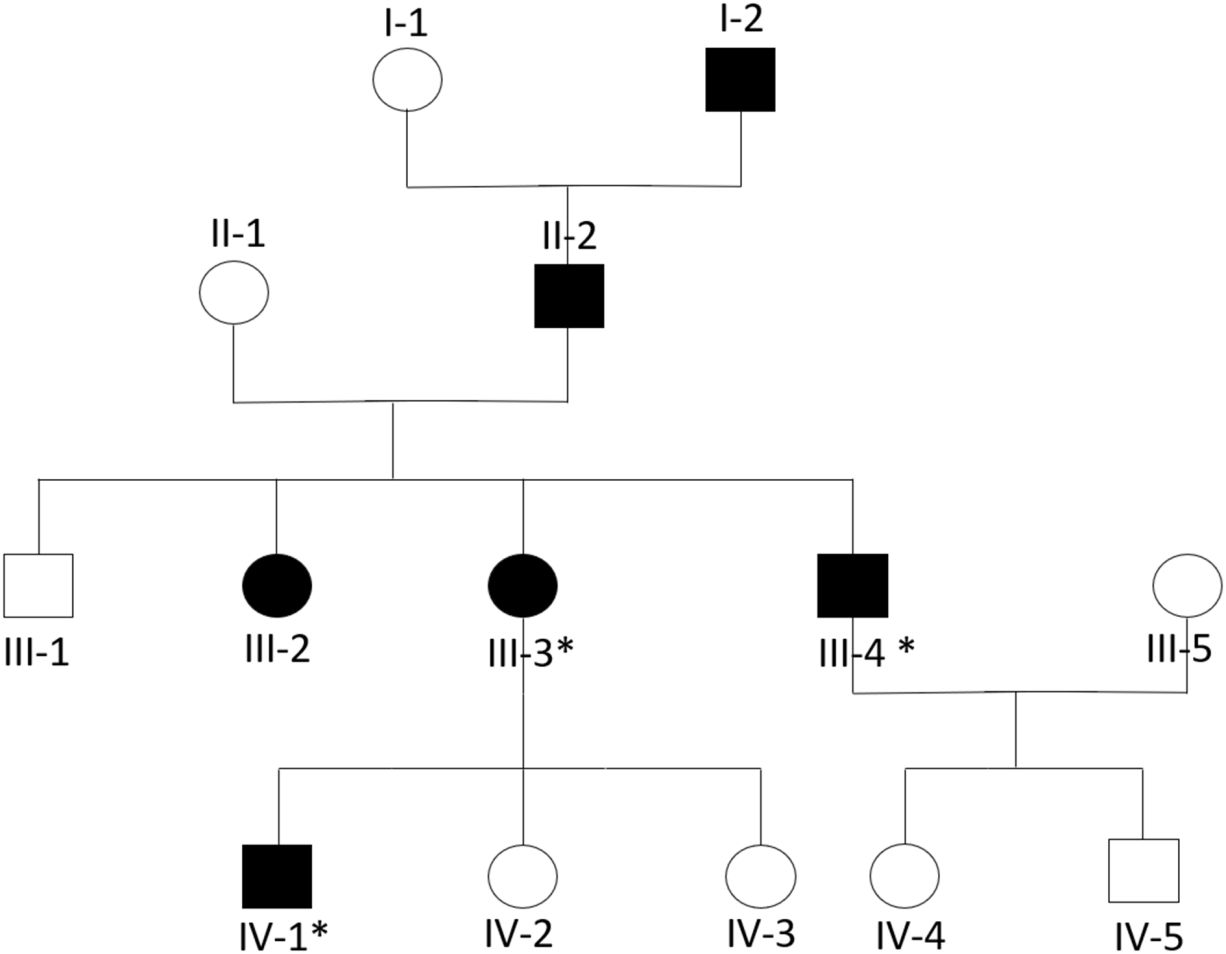
Pedigree. This figure presents the families' pedigree. Persons suffering from cancer are marked black. Subjects tested for the identified *MLH1* variation are marked by an asterisk.

The reported family includes seven additional family members who have suffered from at least one LS‐associated cancer (Figure 1 and Table 1). The age of onset of the first cancer was between 40 and 61 years. The spectrum included colorectal, bile duct, gastric, endometrial, pancreatic, and squamous cell cancer of the nasal skin. IHC and microsatellite analyses of cancerous tissue showed a consistent loss of *MLH1/PMS2* activity in patients III‐2 and III‐4, while panel and exome sequencing in combination with MLPA of DNA derived from blood failed to identify a causal germline variant.

**FIGURE 2:**
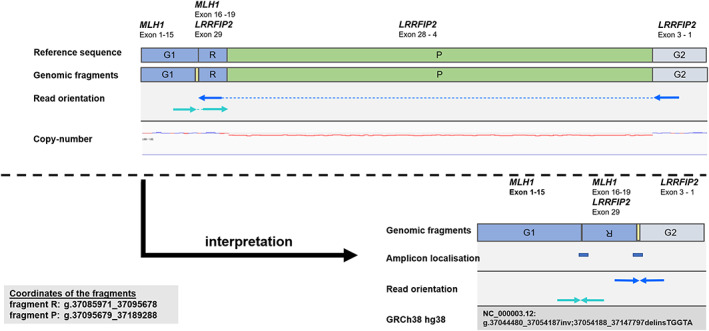
Genomic fragmentation and breakpoint PCR. Panel A presents the information shown in IGV, while panel B illustrates the subsequent interpretation of this information and the position of the breakpoint PCR amplicons. G1 and G2 represent the unaltered genomic fragments. The complex variant includes an inversion of fragment R and a loss of fragment P as well as an insertion of the fragment marked in yellow (TGGTA). The deletion of fragment P was identified solely due to context and reduction in copy number. This genomic variant is leading to two stable in‐frame fusion transcripts that include MLH1 exons 1–15 and LRRFIP2 exon 29 and LRRFIP2 exon 1–3 fused with MLH1 exons 16–19.

GS sequencing was conducted on the genomic DNA of the index patient as described previously (Weisschuh et al., 2021). In total, 1.5 billion reads were generated, and the average sequencing depth of the genome was 62×. *MLH1* was sequenced without any noticeable gaps. No pathogenic or likely pathogenic variant affecting the coding sequence or exonic splicing was identified neither in *MLH1* and *PMS2* nor in *MSH2* and *MSH6*. CNV detection was unremarkable. Subsequent analysis of structural variants indicated a complex structural variant including a paracentric inversion on the p‐arm of chromosome 3 (Figure 2). One breakpoint of the inversion is located in an Alu‐sequence (AluSz6) in intron 15 of *MLH1* (NM_000249.4), and the other is located next to exon 29 of its neighbor *LRRFIP2* (OMIM *614043) (NM_006309.4). The exons 2–28 of *LRRFIP2* were deleted, and an insertion of the nucleotides TGGTA was found (NC_000003.12: g.37044480_37054187inv; 37054188_37147797delinsTGGTA). Subsequent transcriptome sequencing (95 million reads, paired‐end sequencing of 2 × 109 bp, 54× average coverage of coding exons) confirmed two stable in‐frame fusion transcripts that included (1) *MLH1* exon 1–15 and *LRRFIP2* exon 29 and (2) *LRRFIP2* exon 1–3 fused with *MLH1* exon 16–19. We designed primers spanning the breakpoint regions (Figure 2). The targeted sequence was not known to be present in the reference genome. The breakpoint PCR was used to segregate the variant in the patient's family and identified the structural variant was found in the family members IV‐3 and IV‐1. Based on these results, the complex structural variant in *MLH1* was classified as medically relevant. This decision was mainly based on the loss of exon 16–19 of *MLH1* without disruption of the reading frame within one transcript and vice versa the solely expression of these exons within the other. This situation leads most likely to two defective gene products, which contain sections of *MLH1*. Deleterious variants in this region are well established to be causative for Lynch syndrome (Baudhuin et al., 2005; Casey et al., 2005). The variant was not found in public databases and was confirmed by transcriptome sequencing. On principle, we aim for a variant classification according to the ACMG‐Guidelines (Richards et al., 2015). In this case, however, we found them not to be fitting properly, in particular because they do not suggest conclusive parameters for variations like the one presented. In our opinion, an extension of the classification guidelines should be considered, based on the special characteristics of each individual structural variation and the genes it affects.

## 2 DISCUSSION AND CONCLUSIONS

Of all known P/LP variants in HNPCC, *MLH1*, and *MSH2* account for about 70–90% of all deleterious variants (Wu & Vasquez, 2008). Furthermore, 10%–30% of all variants are located in *PMS2* and *MSH6* (Duraturo et al., 2013) and up to 3% in *EPCAM* (Huth et al., 2012). In approximately half of the Amsterdam‐II positive families, no causative genetic variant can be found at present (Park et al., 2002). While causal copy‐number variants are found to be rare, some researchers nonetheless assume them to be one possible explanation for a subset of unsolved HNPCC cases (Rhees et al., 2014). Copy‐neutral variations have been found to be especially rare within the German population (~1%) (Morak et al., 2020). Other explanations for unsolved HNPCC cases may be due to variations affecting regulatory regions (Gazzoli & Kolodner, 2003).

We present a case that shows the advantages of GS with regard to the detection of SVs. A *MLH1* copy‐neutral inversion that involves the genes *MLH1* and *LRRFIP2* was found, which was missed by current standard diagnostic approaches. For *LRRFIP2* (OMIM *614043), no phenotype is listed in the OMIM database. The SV was confirmed by transcriptome sequencing. SVs involving both genes were previously identified in a Portuguese family (Pinheiro et al., 2011). To our knowledge, in the context of HNPCC/Lynch syndrome, a CNV‐neutral rearrangement affecting only two genes, like the one reported in this study, is only rarely identified (Arnold et al., 2020; Momma et al., 2019; Pinheiro et al., 2011; Schneider et al., 2018). We further confirm with our study that transcriptome sequencing from blood can be a useful confirmation for structural variants in HNPCC, as has been shown for other diseases (Yepez et al., 2022).

Besides the reported case, SVs are difficult to classify as standard classification criteria are mostly missing. It would be important, for example, to expand the ACMG criteria to the special features that have to be considered, possibly even gene‐specific rules would be necessary comparable to classification recommendations for single nucleotide variants and small insertions and deletions (Fortuno et al., 2021; Lee et al., 2018). Moreover, the contribution of findings to public databases and identification of similar variants are part of the established procedures in most diagnostic laboratories. However, checking the available data found in UCSC, GnomAD, and decipher at the time of writing, we found only noncomplex SVs altering the CN of LS genes. Although it seems possible to upload more complex SVs (like the one described in this paper) to some of these databases, we found this process to be rather unintuitive. To our knowledge, there is no database that is dedicated to structural variants. Given the expected number of copy‐neutral variants and the apparent absence of these variants, we conclude that these variants are underrepresented. This may either be due to a diagnostic gap or due to problems sharing this information via the established databases.

In our family, the detection of the variant allowed the confirmation of the clinical diagnosis and, more importantly, the identification of patients at high risk for LS‐associated cancers. An intensified surveillance program is offered to all carriers of HNPCC causative variants (Levin et al., 2008). It is also important to identify relatives not carrying the familial variant, and thus without increased cancer risk, to preclude the person from intensified surveillance.

## 3 CONCLUSION

This case report underlines the added value of GS to current standard diagnostic approaches. Complex copy‐number neutral rearrangements are underdiagnosed and could be a relevant factor in LS families. GS ended the diagnostic odyssey of our family and allowed the identification of at‐risk family members. Moreover, GS includes regulatory regions as well as complex genetic markers like polygenic risk scores and offers the opportunity for future reanalysis of data for families without significant findings. With decreasing costs, GS is a competitive and efficient first‐tier diagnostic test.

Using GS, we were able to identify a complex genomic variant that was found to be copy‐number neutral in regard to *MLH1*, and which had been undetected by current standard sequencing approaches. The variant was classified as causative of LS. The case presented had not been solved for several years, despite several institutions using what were then state‐of‐the‐art diagnostics.

## AUTHOR CONTRIBUTONS

*Study conception and design*: Christopher Schroeder, Ulrike Faust and Dennis Witt. *Data collection*: Ulrike Faust, Gertrud Strobl‐Wildemann and Dennis Witt. *Analysis and interpretation of results*: Ulrike Faust, Marc Sturm, Rebecca Buchert, Theresia Zuleger, Jakob Admard, Nicolas Casadei, Stephan Ossowski, Olaf Rieß, Tobias Haack and Dennis Witt. *draft and/or revised manuscript preparation*: Ulrike Faust, Christopher Schroeder and Dennis Witt. All authors reviewed the results and approved the final version of the manuscript.

## CONFLICT OF INTEREST STATEMENT

There are no competing interests.

## CONSENT

All participants consented to contribute to this case report.

## Data Availability

The data that support the findings of this study are available on request from the corresponding author. The data are not publicly available due to privacy or ethical restrictions.
